# Autologous Adipose-Derived Tissue Stromal Vascular Fraction (AD-tSVF) for Knee Osteoarthritis †

**DOI:** 10.3390/ijms232113517

**Published:** 2022-11-04

**Authors:** İbrahim Vargel, Ali Tuncel, Nilsu Baysal, İrem Hartuç-Çevik, Feza Korkusuz

**Affiliations:** 1 Department of Plastic Reconstructive and Aesthetic Surgery, Medical Faculty, Hacettepe University, Altındag, Ankara 06230, Turkey; 2 Department of Chemical Engineering, Engineering Faculty, Hacettepe University, Universiteler Mahallesi, Hacettepe Beytepe Campus #31, Çankaya, Ankara 06800, Turkey; 3 Medical Faculty, Hacettepe University, Altındag, Ankara 06230, Turkey; 4 Department of Sports Medicine, Medical Faculty, Hacettepe University, Altındag, Ankara 06230, Turkey

**Keywords:** adipose tissue derived tissue stromal vascular fraction (AD-tSVF), articular joint cartilage, knee osteoarthritis

## Abstract

Adipose tissue contains adult mesenchymal stem cells that may modulate the metabolism when applied to other tissues. Stromal vascular fraction (SVF) can be isolated from adipose tissue mechanically and/or enzymatically. SVF was recently used to decrease the pain and improve the function of knee osteoarthritis (OA) patients. Primary and/or secondary OA causes inflammation and degeneration in joints, and regenerative approaches that may modify the natural course of the disease are limited. SVF may modulate inflammation and initiate regeneration in joint tissues by initiating a paracrine effect. Chemokines released from SVF may slow down degeneration and stimulate regeneration in joints. In this review, we overviewed articular joint cartilage structures and functions, OA, and macro-, micro-, and nano-fat isolation techniques. Mechanic and enzymatic SVF processing techniques were summarized. Clinical outcomes of adipose tissue derived tissue SVF (AD-tSVF) were evaluated. Medical devices that can mechanically isolate AD-tSVF were listed, and publications referring to such devices were summarized. Recent review manuscripts were also systematically evaluated and included. Transferring adipose tissues and cells has its roots in plastic, reconstructive, and aesthetic surgery. Micro- and nano-fat is also transferred to other organs and tissues to stimulate regeneration as it contains regenerative cells. Minimal manipulation of the adipose tissue is recently preferred to isolate the regenerative cells without disrupting them from their natural environment. The number of patients in the follow-up studies are recently increasing. The duration of follow up is also increasing with favorable outcomes from the short- to mid-term. There are however variations for mean age and the severity of knee OA patients between studies. Positive outcomes are related to the higher number of cells in the AD-tSVF. Repetition of injections and concomitant treatments such as combining the AD-tSVF with platelet rich plasma or hyaluronan are not solidified. Good results were obtained when combined with arthroscopic debridement and micro- or nano-fracture techniques for small-sized cartilage defects. The optimum pressure applied to the tissues and cells during filtration and purification of the AD-tSVF is not specified yet. Quantitative monitoring of articular joint cartilage regeneration by ultrasound, MR, and synovial fluid analysis as well as with second-look arthroscopy could improve our current knowledge on AD-tSVF treatment in knee OA. AD-tSVF isolation techniques and technologies have the potential to improve knee OA treatment. The duration of centrifugation, filtration, washing, and purification should however be standardized. Using gravity-only for isolation and filtration could be a reasonable approach to avoid possible complications of other methodologies.

## 1. Adipose Derived (AD) Tissue Stromal Vascular Fraction (tSVF): Perspective of Plastic Reconstructive and Aesthetic Surgery

Adipose tissue is complex and heterogeneous [1]. It does however contain a high population of regenerative cells including adult mesenchymal stem cells (MSCs) that can be retrieved with minimal morbidity [2]. Micro-fat is anti-apoptotic, anti-catabolic, anti-fibrotic, anabolic, pro-chondrogenic, and immune modulatory [3,4,5,6]. Nano-fat injections into the lipofilling area are recommended for skin regeneration [7]. Stromal Vascular Fraction (SVF) alone or in combination with Platelet-Rich Plasma (PRP) was used to stabilize the transplanted adipose tissue in reconstructive surgery [8] even though 22% apoptosis and 11% necrosis were observed in these cells [9].

Fat grafting has been widely used for volume-filling purposes in plastic, reconstructive, and aesthetic surgery since the late 1890s. Conventional fat harvesting, grafting, and injection methods are suitable to reconstruct large amounts of tissue volume losses such as in breast augmentation, the treatment of radiation damages, and restoration of scar tissues. For finer areas such as facial injections, however, smaller cannulas are used to harvest the fat tissue [7]. This approach is named as micro-fat grafting. Further processing of the micro-graft has created an even finer material, which is named as nano-fat grafting. Nano-fat grafting is currently used in infraorbital regions or for intradermal injections. Nano-fat induces matrix regeneration and remodeling, which is a reliable method in regenerative and restorative medicine. A recent approach was to separate cells mechanically and/or enzymatically from the fat tissue that contains MSCs. Mesenchymal stem cells remain relatively stable in the micro- and the nano-fat graft environment as well as in SVF [7]. Cell Counts in Macro-, Micro-, and Nano-Fat per 100 mL of Lipoaspirate with a standard macro-fat, multi-perforated, and micro-fat with multi-perforated and emulsification nano-fat cannulas ranged between two to three million, 100 to 200 thousand and five to 6.5% for SVF, CD34+ cells in fraction and CD34+/SVF %, respectively [7].

## 2. Structure and Function of Articular Joint Cartilage

**Figure 1:**
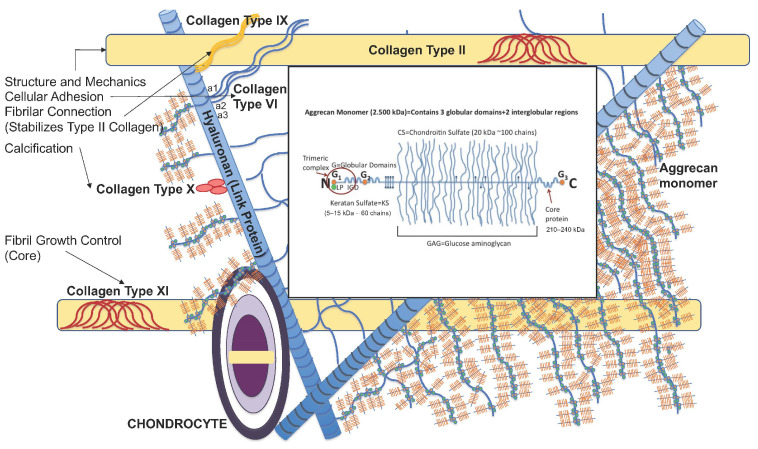
Structure of articular joint cartilage.

Articular joint cartilage is a major component of the synovial joints that allows movement through lubrication. It contains one type of cells that is named as chondrocytes. The rest of the articular joint cartilage is mostly made of the extracellular matrix (ECM) and lacks blood and lymph vessels and nerve endings. Chondrocytes are therefore nourished by the synovial fluid, and their primary function is to maintain or restore the ECM. The ratio of chondrocytes to the ECM is low when compared to other tissues [10]. In case of injury, therefore, the regeneration ability of articular joint cartilage is very limited [11]. The ECM structure consists of a main binding protein called type 2 collagen. Hyaluronan (HA) is the link protein, and several Glycosaminoglycan (GAG) chains are attached to it by their N terminal. Chondroitin Sulfate (CS) and GAG binds to and preserves large amounts of synovial fluid for lubrication. About 80% of the wet weight of articular joint cartilage is off the synovial fluid [12,13]. In its structure, apart from type 2 collagen, there are other proteoglycans named as aggrecan, decorin, biglycan, and fibromodulin. Other collagens such as type III, VI, IX, and XI function for the homeostasis of articular joint cartilage. Mechanical loads are also important to maintain its structure and function (Figure 1) [14]. The ratio of CS and Keratan Sulfate (KS) may change with aging and degeneration [15]. Articular cartilage is of four layers according to its structural arrangement of chondrocytes and ECM. Cells are fibrillar, and the ECM fibers are parallel to the surface in the layer closest to the articular surface. The penetration of nutrients to its deeper layers is regulated by this layer and by mechanical forces. Injury at the surface layer triggers inflammation and initiates an immune response [14]. In its central and deep layers, the cells are arranged vertically within their lacunae, and the orientation of the fibers is perpendicular to the surface. The middle layer absorbs shear stress generated at the joint movement, and the deepest layer separates joint cartilage from the subchondral bone [16].

## 3. Definition and Treatment of Osteoarthritis (OA)

Primary osteoarthritis (OA) [17] is defined as a chronic disabling disease with a familial genetic predisposition [18], whereas secondary OA is frequently initiated by excessive joint loads or overuse that cause wear and tears at the surface of the articular joint cartilage [19]. Primary OA is mostly related to overweight, aging, and heredity. Obesity is the greatest modifiable risk factor for OA. A study [20] reported that individuals with a Body Mass Index (BMI) higher than 30 kg/m^2^ were 6.8 times more likely to develop knee OA than normal-weighing participants. Injury at the meniscus, capsule, and ligaments may trigger joint cartilage degeneration in secondary osteoarthritis. Inflammation, degeneration, and regeneration at the tissue level of the articular joint cartilage, the subchondral bone, and all other components of the joint (https://www.cdc.gov/arthritis/basics/osteoarthritis.htm; accessed on 3 October 2022) are common findings. This cascade continues even if the primary injury site is repaired. Secondary OA may also develop after septic arthritis and may accompany some developmental and hereditary diseases and/or disorders. Studies [21,22] defined genetic, estrogen-dependent, and age-dependent osteoarthritis. The disease is most common in the ankle, the knee, the hip, the spine, and the hand joints. Symptoms of pain, swelling, deformity, and loss of function are prominent in patients. Radiological findings are narrowing of the joint space, osteophyte formation, sclerosis, and subchondral cyst formation [23].

Osteoarthritis affects 33.6% of the population over the age of 65 and 50% of the population over the age of 75 years (https://www.cdc.gov/arthritis/basics/osteoarthritis.htm; accessed on 3 October 2022). The incidence is reported as 203 per 10 thousand people per year [24]. The 2019 EPISER study [25] reported that OA is the fourth most common cause of disability in Spain, with five million individuals affected by the disease. The disease is at the top of the Global Years Lived with Disability (YLD) rankings [26]. Osteoarthritis also has the highest rate after low back pain in the calculation of Disability Adjusted Life Years (DALYs) [27]. In addition to disability and morbidity, an increase in mortality is also observed with OA [28].

**Figure 2:**
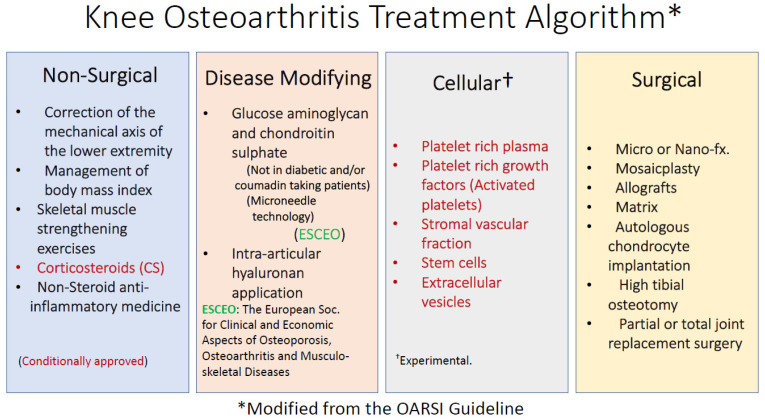
Knee osteoarthritis treatment algorithm (Modified from OARSI).

The major problem in the clinical treatment of OA is the low healing potential of the articular joint cartilage due to lack of vascularity and hypocellularity. Joint replacement surgery for the knee and the hip joints accounts for 1.0–2.5% of developed country budgets [29]. In 2009, the total cost of knee and hip replacement surgery for OA patients was USD 42.2 billion [30]. Revision of the implant is inevitable once a joint is replaced at a younger age [31]. For this reason, it is of great importance to postpone joint replacement surgery by modifying the course of the disease. Non-surgical treatments for osteoarthritis include reducing the BMI by exercise and a healthy life style, increasing the strength of periarticular skeletal muscles with minimal joint loading exercises, reducing pain with non-steroidal anti-inflammatory medicine, and increasing the joint range of motion and function with physical medicine and rehabilitation methods (Figure 2) [32,33]. In addition to conventional OA treatment modalities, there are also disease-modifying approaches.

Glucosamine (GAG) and chondroitin sulfate (CS) may reduce inflammation, slow-down OA progression, and help to the regeneration of articular joint cartilage [34]. GAG and CS also supported synoviocyte growth. They reduced NF-kB levels and downregulated COMP biomarkers. The amount of daily oral intake of such supplements and their absorption through the gastrointestinal system however could vary between individuals. However, diabetic seniors or patients on coumadin treatment cannot use these supplies [35,36,37]. Hyaluronan, the link protein of articular cartilage, is commonly injected into the joints [38]. Hyaluronan injection decreased pain during walking and stair climbing in knee OA patients [30]. Patients however do not consider diet and exercise as a treatment option despite the recommendation of their physician. By this approach, the age-dependent deterioration of CS and its conversion to KS is partially regulated [39]. The basic approach in the regeneration of the osteochondral defects is therefore the restoration of the original articular joint cartilage with type 2 collagen.

**Figure 3:**
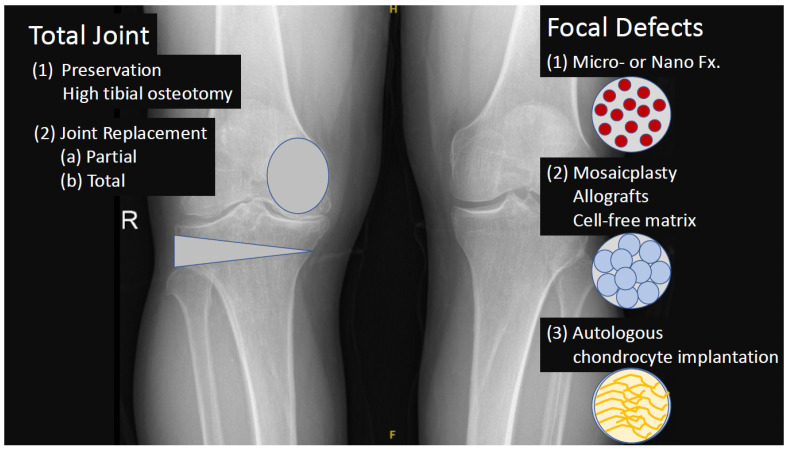
Surgical methods for treating articular cartilage defects and osteoarthritis.

Secondary OA can occur if osteochondral defects or articular cartilage is not recognized and treated on time. The micro-fracture or recently the nano-fracture technology, which improves subchondral bone vascularity, is practiced arthroscopically [40,41,42]. Since the factors coming from the blood stream into the joint however cause type I instead of type II collagen synthesis, a preventive barrier between the articular cartilage layer and subchondral bone is preferred. Reduced pain and an increase in function were observed in early follow-up with such surgical procedures [43]. Creating nano-fractures instead of micro-fractures in the subchondral bone in defects smaller than four cm^2^ is a new approach [44]. Micro- and nano-fracture techniques are often combined with cellular regenerative treatments such as PRP, SVF, and/or MSCs injections with or without scaffolds. Mosaicplasty is another articular regeneration approach by replacing healthy cartilage and bone plugs’ non-load-bearing areas to the damaged area. Possible side effects of mosaicplasty and a comparison with allograft were recently published [45], and good results were reported at the mid-term follow-up. Autologous chondrocyte implantation is a novel technique that involves harvesting cartilage tissue from the non-load-bearing area of the joint, transferring the tissue to the laboratory environment, separating the cells by processing, and then applying the chondrocyte-like cells reproduced in the culture medium to the patient. The most important problem of mosaicplasty and autologous chondrocyte implantation is the possible negative side effects at the donor area. Mesenchymal stem cell application alone, with matrix or with matrix plus induction factors, is a current experimental approach in osteochondral defect repair. Stromal vascular fraction is also currently used in osteochondral defect repair since a portion of the stem-cell-containing compound is harvested from the adipose tissue (Figure 3).

In the early stages of OA, besides the articular cartilage and subchondral bone, the synovium, infra-patellar adipose tissue, ligaments, and joint capsule are also affected. Molecular communication between cells in these tissues and adipocytes, mesenchymal stem cells, M1 and M2 macrophages, NK cells, Mast cells, B and T lymphocytes, and monocytes occurs via IFN, TNFα, adipokine, fatty acids, and MMPs [46]. Humoral homeostatic mechanisms try to balance intra-articular inflammation through the synovium and fat-derived stem cells at this stage. Early OA is an insidious phase of the disease since patients are either asymptomatic or present with only reduced clinical symptoms [47]. A clear definition of the early OA phase is important from two points of view: it could allow one to identify patients before advanced degenerative phases, which definitely contraindicates the current regenerative treatments [48]. OA-specific biomarkers such as IL-6, IL-8, matrix metalloproteinase (MMPs), cartilage oligometric matrix protein (COMP), tumor necrosis factor (TNF), microRNAs, growth differentiation factor 11 (GDF-11) that could also be detected in the serum or synovial fluid in patients with suspected OA would be useful for early diagnosis, the examination of the disease progression, and the development of targeted therapies [49].

Osteophyte formation is an early event in OA; considerable deformity based on bony enlargement such as muscle atrophy and joint effusion are more characteristic of advanced OA stages. The changes in the subchondral bone in early OA start with an undulation of the subchondral bone—cartilage interface, a progressive increase in the thickness of the subchondral bone plate, and a remodeling of the subarticular spongiosa. Structural changes in early OA chiefly occur in the articular cartilage and the subchondral bone, although early OA affects all other structures of the knee joint, such as the synovial membrane, the menisci, the joint capsule, ligaments, muscles, and the infrapatellar fat pad (IFP) [50]. If IFP macrophages are activated by a variety of interleukins and interferons secreted from other resident and infiltrating immune cells and adipose cells within the IFP, they begin secreting vast amounts of pro-inflammatory cytokines, catabolic factors, and adipokines, and, with prolonged periods of time, the IFP can also release pro-fibrotic mediators such as CTGF that may contribute to KOA progression. At the same time, the description of the presence of mesenchymal stem cells (MSCs) as perivascular cells within the IFP (IFP-MSC), exhibiting immunomodulatory, anti-fibrotic and neutralizing activities over key local mediators, has promoted the IFP as an alternative source of MSCs for cell-based therapy protocols [46].

Too advanced tissue damage might negatively influence the potential benefit since knee joint tissues and their biological response could be already compromised by anatomical structural changes not addressable with simple injective procedures. In the initial stage of the disease, with no clear lesions or associated abnormalities requiring to be addressed surgically, local injective treatments might have a higher potential to influence the joint microenvironment positively and lead to a clinical improvement [50].

Adipose progenitor cells may regulate the homeostatic mechanisms of tissues [51], and ADMSCs co-cultured with hyaluronan may mediate secretomes of inflammation [52]. Macrophage polarization and subsequent fibrosis leading to adhesion are observed in these tissues if the inflammation is not stabilized. The regenerative potential of tissues is limited, and catabolic mechanisms may be favored to lead to destruction. While M1 macrophages increase tissue damage by triggering IL-1b, IL-12, and TNFα, anti-inflammatory molecules from the SVF such as IL1Ra, IL-10, TGFb, and Arg1 initiate repair by maintaining the balance in the tissue. Oxidative stress occurs with age along with inflammation [53]. Oxidative stress leads to dysfunction in cellular mitochondria. This disrupts the electron transport chain proteins within the mitochondria. Reductions in electron transport chain proteins reduce adenosine in the extracellular matrix. Adenosine in the extracellular matrix prevents OA-induced phenotypic changes. Adipose-tissue-derived cells found in the SVF injected into the joint space and functioning as MSCs interact with chemokines such as CXCR4, integrin, selectin, and vascular cell adhesion molecule 1 and transform into chondrocyte, as well as regenerating the articular cartilage by preserving adenosine in the intercellular space with paracrine effects [54]. It was shown that cells in the SVF exert their anti-inflammatory effects through TNFα and TGFb and also stimulate HIF and IGF-1 [55]. The stromal vascular fraction also contains anti-apoptotic bodies and exosomes [56] rich in glycolytic adenosine triphosphate and micro-vesicles. They also have paracrine effects.

## 4. Adipose Derived Stromal Vascular Fraction (AD-SVF)

Regenerative medicine defines the regeneration of tissues and organs lost by trauma or disease in their original form using tissue engineering, cellular modalities, and active signal molecules [57,58,59]. Personalized cellular and/or precision medicine was recently used as a regenerative medicinal approach. Cells obtained from the original tissue and/or those that can convert to that specific tissue such as stem or induced pluripotent stem cells can be used for personalized or precision medicine.

Adipose derived stromal vascular fraction (AD-SVF) is mechanically and/or enzymatically obtained from fat tissues [60,61]. Cells of the AD-SVF are not proliferated in cultures, and they represent a collection of heterogeneous cells and tissue fragments. Stromal vascular fraction contains paracrine factors that stimulate and enhance endogenic regenerative pathways [60,62]. Its biological and biomolecular action mechanisms include stimulation of angiogenesis, immune modulation, cell proliferation, and differentiation and extracellular matrix functionalization [63]. Isolation of AD-tSVF from the abdominal fat tissue is almost always possible in all patients. The amount of fat tissue for about 3.0–6.0 mL AD-tSVF requires about 60 mL lipoaspiration material. Well-trained athletes with very few amounts of abdominal fat would not be good candidates for AD-tSVF applications [64]. Patients with secondary arthritis, medical conditions that precluded an anesthetic procedure, psychiatric disorders, history of cancer, pregnancy, coagulopathy, signs of infection or syphilis- or HIV-positive serological results, knee joint surgery, and intra-articular injection may not be a proper choice although there were no serious adverse effects reported in appropriately selected patients [65,66,67]. Cells and tissue fragments obtained from the adipose tissue are experimented to regenerate tissues related to coronary heart disease, peripheral vascular diseases, dermatopathy, chronic wounds, ischemia [68], diabetic foot ulcers, fistulas, liver fibrosis, lipodystrophy, Burger disease, and radiation-related ulcers [57,69,70]. Mesenchymal stem cells obtained from the adipose tissue are effective in treating knee OA; however, their action mechanism, dose, repetition, application duration, and evaluation parameters need to be determined [71,72]. Clinical trials and applications (clinicaltrials.gov; accessed on 12 August 2022) related to AD-SVF are alopecia, OA, lymphedema, pressure ulcers, Chron disease, type II diabetes, erectile function disorders, multiple sclerosis, chronic obstructive lung disease, high tibial osteotomy, micromastia, dermal grafts, systemic sclerosis, ischemia, peripheral vascular disease, rheumatoid arthritis, systemic lupus erythematosus, and non-curing dermal lesions (Table 1) [60].

The World Health Organization (WHO) defined chronic disorders including OA and osteoporosis (OP) as the most important health condition in an aging society after cardiovascular and neurodegenerative diseases. Osteoporosis is determined with bone loss, and OA is defined with pain, loss of function, and deformity that cause morbidity and mortality. Loss of motion and function in OA increases the mortality risk about 6% when compared to age-matched controls [73]. The percentage of the population above the age of 65 is 27% in Japan [74]. Osteoarthritis is therefore defined as the most important chronic disorder in that country. High-energy trauma related to road traffic accidents, gun-shot wounds, and workplace injuries causes the loss of tissues and organs. Stromal vascular fraction is used to treat such diseases and disorders [75,76]. Cellular therapies are more frequently used to decrease inflammation and stimulate the regeneration in systemic metabolic diseases such as OP and painful other musculoskeletal disorders including skeletal muscle, tendon [77], ligament, bone, and joint injuries [76]. Mesenchymal stem-cell-based therapies aim to decrease inflammation and trigger regeneration by synthesizing cytokines and active signaling molecules, whereas they may also convert to the cells of the target tissue and stimulate regeneration by their paracrine effects [78]. Such an approach in well-vascularized tissues breaks the chronic inflammatory cascade and stimulates neo-vascularization [79,80,81]. There is also an increasing public interest in MSCs’ treatments for the OA of the knee and hip joints [82]. That study revealed that patients and their relatives increased their internet search from 54.4–78.1% on cellular treatments for their chronic disorders. AD-tSVF that contains active molecules and cells and requires a minimal invasive approach is defined as an optimum tissue regeneration product for the treatment of OA [23,83]. A review [71] indicated that SVF may decrease joint degeneration and increase articular joint cartilage regeneration. This approach further decreased pain and improved function in patients. Stromal vascular fraction products in the USA are regulated according to Food and Drug Administration (FDA) guides 351 (a) published in August 2014 (https://www.fda.gov/media/89049/download; accessed on 2 October 2022) and regulation 361 published in 2018. Such products are evaluated according to Medical Device Directive 93/42/EEC in the European Union. The International Federation for Adipose Therapeutics and Science (IFATS) (https://www.ifats.org/; accessed on 3 October 2022) published an opinion letter for the FDA-2014-D-1856 regulation that was published in 2016. The International Society for Cell & Gene Therapy (ISCT) (https://isctglobal.org/; accessed on 3 October 2022), together with the IFATS, supported the development of the SVF concept [84]. The FDA-2014-D-1584 regulation defines the application of SVF at the same time of the harvesting of the adipose tissue that is relevant to the FDA-2014-D-1856 regulation. The FDA-2014-D-1856 regulation, on the other hand, refers to human cells, tissues, cellular, and tissue-based products obtained from the adipose tissue. This regulation is also related to the FDA-2014-D-3581 regulation, which is on human cells, tissues, cellular, and tissue-based products. Item #351 of the FDA regulation requires pre-marketing approval for medicine, medical devices, and biological products, whereas item #361 that focuses on homologous, minimal manipulative cells and tissues does not. Homologous and minimal manipulative concepts are therefore defined in items 21 CFR 1271.3 (c) and CRF 1271.3 (f), respectively. According to these regulations, adipose tissue is not an endocrine organ; however, it may present metabolic activity through protein synthesis. Angiogenic stimulation and tissue regeneration can be regulated by the cytokine release from this tissue. Cell products manipulated in the laboratory are defined as Advanced Therapy Medicinal Products (ATMP) in Europe, and they are under the regulation of EMA/CAT/852602/2018. SVF obtained and applied at the same time and in the same procedure is therefore favored recently. SVF products in Turkey are covered by the Medicinal Product Regulation #27957 published in April 2011. According to this regulation, obtaining the tissue in operating theater conditions and applying it to the disorder and/or injury site at the same session after minimum manipulation by using disposable medical devices if possible are recommended. This approach is defined as Injectable Tissue Replacement and Regeneration (ITR2); however, clinical ethical board permission is required if the tissue will be used for the regeneration of another tissue.

Stromal vascular fraction is prepared by mechanical extrication and/or enzymatic digestion [85]. Stem-cell-like cells of the adipose tissue [86] and progenitor cells [87] were defined in the years of 2002 and 2008, respectively. In 2008, an in vivo study confirmed the decrease in pain and increase in range of motion in 14 beagle elbows [88]. ADMSCs were safe and presented no side effects in a clinical study [89] in 18 knees with knee OA. A team from South Korea applied SVF, which was enzymatically prepared, and PRP to the knee joint of their OA patients [90] that decreased pain and improved function. Platelet rich plasma promoted ADMSCs’ proliferation and chondrogenic differentiation [91,92]. The same group further published [93] that SVF was safe and effective in 91 patients. Swelling was however common in injected joints. This approach was also used in three patients to treat chondromalacia patellae [94]. Findings of these studies were in line with a previous in vivo experiment that combined SVF and PRP proving that this approach was promising for articular joint cartilage injury treatment [95]. Another in vivo study [96] enzymatically digested their ADMSCs using the Celution (Cytori Therapeutics, Inc., San Diego, CA, USA) system and repaired the chondral and subchondral defect in hyaline-rich cartilage in 12 months.

The up-to-date definition of SVF is described by IFATS and ISCT. Safety of ADMSCs and SVF in knee OA after 12–24 months of clinical follow-up was covered in a review [97]. The Korean Food and Drug Administration (KFDA) approved the use of SVF in OA when harvested in a medical center by a medical doctor on the same day with minimal manipulation [98]. The KFDA licenses products as medicine when obtained cells are transferred to the laboratory for further manipulation and proliferation [53]. The regenerative medicine safety act in Japan activated SVF utilization in 2014 [74]. Mesenchymal stem cells attach to the bottom of culture plates, express CD73+, CD90+, CD105+, CD11b-, CD14-, CD19-, CD45-, CD79a-, and they differentiate to osteocytes, chondrocytes, and adipocytes (https://www.isctglobal.org; accessed on 3 October 2022). SVF is marked with the ADMSCs, CD34+, CD45-, CD235a-, and CD31- phenotype [99]. Surface markers CD9+, CD10+, CD29+, CD34+, CD44+, CD49+, CD54+, CD55+, CD59+, CD73+, CD90+, CD105+, CD106+, CD117+, CD146+, CD166+, HLA1, fibronectin, endomusine, ASMA, vimentin, and collajen1 are further SVF indicators [79,100]. ADMSCs, CD11b-, CD13-, CD14-, CD19-, CD29-, CD31-, CD79-, alfaCD80-, CD117-, CD133-, CD144-, HLA-DR-, c-kit-, MyD88-, STRO-1-, Lin-, and HLA2- surface markers on the other hand are not SVF indicators [61,78].

Preserving the lubrication function of the articular joint cartilage is mostly determined by genetics and mechano-biology. Loss of function is a major clinical finding in OA. SVF applied to the joint in OA decreases inflammation, apoptosis, and fibrosis [101]. SVF application is minimally invasive, and patient comfort during this application is at acceptable levels [102]. Administration of cells and active molecules directly to the tissue is the other advantage of this application. After local anesthesia, liposuction, processing, and application of isolated AD-tSVF should be immediately performed during the same session [103,104].

A study reported a clear tendency towards a reduction in vitality and a change in cell composition between 8 and 24 h of storage of SVF. Both prolonged storage time and increased temperature during lipoaspirate storage negatively affected the quality of the obtained SVF. The results suggest that lipoaspirate should be stored for no longer than 24 h at 4 °C to maintain the optimal quality for the isolation of SVF and the expansion of ASCs [105,106].

Stromal vascular fraction contains the platelet-derived growth factor (PDGF) [79] and vascular endothelial growth factor (VEGF) that activate the migration of endothelial cell precursors to the regeneration site [107]. Matrix remodeling is initiated and managed by fibroblast growth factors (FGF)-2, -6, and -7, its receptor FGFR3, fibronectin, and integrins a5, a11, and b1 [79]. It increases TIMPs-1, -2, -3, and -4 metalloproteinases and inhibits MMP-1, MMP-3, MMP-13, and MMP-28 while intensifying ADAMTS-4 and -5 by regulating the joint molecules (Table 2) [79,108]. ADMSCs applied to 18 patients with severe knee OA decreased systemic inflammation [109]. Intraarticular SVF administration in OA increased GAG in hyaline cartilage [10]. The ideal dose of SVF however is not determined yet [110]. Applying SVF together with PRP and/or after the micro- or the nano-fracture procedure may alter the clinical outcome. Intraarticular SVF application has decreased pain, an improved joint range of motion, and a decrease in the radiological size of the chondral defect [72]. The procedure caused mild pain and minimal swelling at 48–72 h in 26 joints of 17 advanced knee OA patients. A follow-up procedure for patients with an FDA-approved device was defined [111]. Pain decreased and function improved when SVF was administered together with PRP in 350 knee and hip OA patients [112]. A total of 80 male and 65 female patients between the ages of 15 and 85 received SVF after collagenase digestion [62]. Adverse effects with 5 mL 8 × 10^6^ cell count in the joint, ligaments, tendons, and skeletal muscles were not observed. Three to five injections were undertaken after the SVF was frozen in liquid nitrogen.

There is a trend to shift to mechanical extraction of SVF due to the potential side effects of enzymatic digestion (Table 3). Mechanical extraction of SVF is also named as micronized tissue fragments as stromal-like tissues remain in the elution. The heterogenous cell and tissue composition of SVF in micronized tissue fragments determines its function. Surface markers for stem cells are two times higher after mechanical separation [113] although the cell number and density increase after enzymatic digestion [114]. The success in separation may change from the brand and batch of the enzyme. A xenogeneic immune reaction can be observed in animal-originating enzymes. The enzymatically derived SVF targeted the synovial tissue, whereas the mechanically separated SVF targeted the joint cartilage in rabbit experiments [102,115]. Enzymatically separated ADMSCs on the other hand attached, proliferated, and produced a higher number of fibroblast-like cells in cultures when compared to the mechanically separated SVF [116]. ADMSCs applied after bilateral meniscectomy were safe in rabbit knees [117]. Stromal vascular fraction was also combined with hyaluronan-based scaffolds with good results in animal experiments [118].

The enzymatic [119] and non-enzymatic mechanical separation methodology of SVF is well defined [120,121]. Preserving the cell-matrix integrity by using mild mechanical forces is another advantage of AD-tSVF. Tissue integrity protects cellular destruction and implements a biophysical support. Bacterial contamination is also less in AD-tSVF. The genomic stability is higher; however, these products cannot be delivered systematically through the blood route [122]. Cell density in mechanical separation is about 5.0%, whereas it increases up to 25.9% when combined with enzymatic digestion [123]. Steps of SVF separation can be summarized as (a) liposuction, (b) mechanical separation or fraxination, (c) initial filtration, (d) washing, (e) final filtration, (f) SVF and adipose graft harvesting, and (g) cell counting and/or characterization (Table 4). Centrifugation and/or incubation can or cannot be included into any of these stages.

The cell number increases several folds in enzymatic isolation, whereas the cell surface markers are better expressed when enzymes are not used [124]. When compared to bone marrow concentration, AD-tSVF had less nucleated cells [125]. Their colony-forming unit frequency and adherent cell numbers were however higher. Exosome content was also higher in the non-enzymatic processed human adipose tissue [126]. Using enzymes for SVF isolation however may cause a severe inflammatory response, massive lymphocyte infiltration, neovascularization, and cartilage destruction after in vivo administration [127,128]. AD-tSVF was reported to be safe, less costly, and less time consuming [129]. This however contains more mononuclear blood cells and less progenitor cells.

Washing, shaking, vibration, and centrifugation are common methods of mechanical SVF extraction [130]. Advantages and disadvantages of these mechanical extraction methods were not studied yet. The speed and duration of these modalities were also not determined precisely. Disruptive forces during these approaches and during filtration may lead to a fewer number of active cells. Colony formation rates were higher in SVF when they were compared to cells aspirated from the bone marrow. All cells independent from their origin need to be screened for their CD31, CD34, CD45, CD73, CD90, and CD105 surface markers in flow cytometry before application [130].

Stromal vascular fraction also provides an environment where MSCs can interact and stimulate regeneration [131]. In 2018 [132] and 2019 [122], enzymatic separation of SVF was named as cellular cSVF and mechanical separation as tissue tSVF, respectively. Tissue processing instead of defining them as separation could be a better statement for SVF preparation. AD-tSVF was as effective as AD-cSVF. AD-cSVF contains adipocytes, pre-adipocytes, fibroblasts, pericytes, macrophages, and MSCs. All these cells, when applied clinically, stimulate the repair and regeneration of tissues. They exaggerate a paracrine effect that is more important. A systemic review published in 2020 [133] evaluated the AD-tSVF outcomes of 1443 patients cited in 34 articles. Eight of the studies were on dermal applications. Five of them covered the repair of skin lesions. Six articles focused on OA, two on tendinopathy, one on the temporomandibular joint, one on androgenic alopecia, one on perianal fistula, three on migraines, and one on vocal cord adhesion. Mild complications were recorded in 3.5% of the patients, and they were only at the lipoaspiration site. Different adipose tissue isolation and separation procedures were used in all these studies; however, overall, good results were achieved in a limited number of patients during short-term follow-up. A study [131] combined the micro-fracture procedure with SVF in 20 patients and reported successful outcomes at the 12-month follow-up. The AD-tSVF procedure is safe, legal, and easy to apply. The GAG content in MR increased in certain locations of the joint cartilage in 32 knees of 17 patients after SVF application [134]. Thirty-nine knee OA patients were followed up with low- and high-dose SVF administration [135]. Pain decreased and function increased in the high-dose SVF-applied group, whereas MR showed no difference. A retrospective study in 2019 [74] compared ADMSCs with SVF after six months of administration. Pain decreased and function increased earlier in the ADMSCs-administered group, whereas swelling, which was higher in the SVF group in the early stages of treatment, disappeared equally in both of the groups at the end of the follow-up period. ADMSCs were presented to reduce the pain after the successful repair of a torn meniscus in a 32-year-old female patient [136]. Pain decreased and function increased after injection. PRP and CaCl_2_ were additionally injected into the joint space after three and seven days in that study. On day 14, dexamethasone, and, on day 28, PRP and CaCl_2_, were re-injected. Multiple injections by combining MSCs, PRP, CaCl_2_, and dexamethasone could however affect the evaluation of outcomes. A controlled study design could help the better understanding of each of these applications. A study [137] administered enzyme-derived ADMSCs to 18 knees of 33 OA patients after the microfracture procedure. The visual analogue score (VAS), WOMAC, and bone marrow edema in MR decreased at 12 and 24 months, whereas Lysholm and Outerbridge scores in MR increased in these patients. Another study [138] applied ADMSCs to three joints of two patients. The visual analogue score and KOSS improved after three and six months. Regeneration of the joint cartilage was also observed during the second-look arthroscopy. Mesenchymal stem cells derived from micro-fragmented adipose tissue are well characterized [139]. Pain decreased and function improved after a year in 20 OA knees after intraarticular micro-fragmented fat tissue was applied [140].

AD-tSVF was implemented to 38 patients’ knees after chondral procedures [141]. Good results were attained after a year. In another study [142], pain decreased and function improved in 26 knees of 13 patients. Intra-articular AD-tSVF administration was safe and effective in 10 out of 28 patients with knee articular cartilage degeneration [143]. Pain decreased and function increased in 11 knees of six OA patients after SVF application [144]. Side effects related to this application in a month of follow-up were not observed. A decrease in pain and improvement in function after autologous SVF application were also confirmed in 30 knee OA patients [145]. In 2017, a group divided 30 knee OA patients with a Lawrence score of 2 and 3 into the placebo control and arthroscopic micro-fracture, SVF, and PRP application groups [146]. Pain decreased and function improved at the 18 months follow-up in the treatment group. Another research group [134] administered autologous micro-fragmented fat tissue into 35 knees of 17 knee OA patient the same year. They assessed joint cartilage using MR. They furthermore evaluated plasma and synovial fluid IgG, plasma glycan profile, and N-glycan release. Radiology presented joint axis recovery and joint cartilage recovery by gadolinium MR. Glucose aminoglycan changes however were not significant. Another study [147] combined SVF and PRP in ten knee OA patients and presented the decrease in pain and improvement in function. Joint cartilage thickness increased about 0.2 mm in six of these patients, whereas it decreased in two and remained constant in the other two patients. In line with that study, another study [146] published an increase in joint cartilage thickness after micro fracture combined with SVF and PRP application in 15 knee OA patients at an 18 month follow up. Pain decreased and function improved concomitantly in this study. Adding musculoskeletal exercises to SVF and PRP treatment improved the outcomes in a case series [148]. Platelet-rich plasma increased the growth and motility of ADMSCs and controlled the secretory function of these cells [149]. ADMSCs were applied to ankle joint varus osteoarthritis patients after supra-malleolar and sliding calcaneal osteotomies [138,150,151]. Good results were reported after a second-look arthroscopy. SVF was applied to 50 ankles of 49 patients [151]. Outcomes also evaluated using MR were promising even in older-aged and large-sized lesions. A similar approach was used after ablation of the disc by radiofrequency in a 43-year-old patient [152]. A 33-year-old patient received SVF after joint cartilage injury due to skiing [153]. Pain decreased, function improved, and the patient returned to skiing after 30 months. Autologous lipoaspirates are prepared as injectable active scaffolds for a single-stage repair of cartilage defects [154]. A case with non-responsive knee pain with OA and concurrent meniscal disease was treated with autologous micro-fragmented adipose tissue [155]. Twelve patients with lateral epicondylitis were treated with allogenic ADMSCs in a pilot study [156]. Adverse effects were not observed through 52 weeks of follow-up. Elbow pain decreased; performance scores increased; and the tendon defect area measured by ultrasound improved in that study. Second-look arthroscopy is a method to evaluate the outcomes of SVF applications. A total of 60 knees of 56 patients were retrospectively evaluated after SVF application [157]. Encouraging outcomes were related to the patient weight and size of the lesion. A total of 16 out of 30 knee OA patients between 65 and 80 years of age underwent second-look arthroscopy after 4.0 × 10^6^ SVF cell injection [158]. Findings revealed a decrease in pain, improved function, and cartilage healing. Quantifying second-look arthroscopy is however challenging. New methods are needed to demonstrate the lubrication function of the repaired tissue. Micro-fragmented fat tissue was also used in a patient to regenerate oral bone and soft tissues [159]. A total of 120 patients undergoing orthognathic surgery benefited from micro fractured and purified adipose tissue grafts aesthetically [160]. Patients were satisfied, and complications was not observed. Twenty-one OA grade II and III patients were treated with SVF and PRP [161]. Pain decreased and joint functions and MR findings improved after treatment. Side effects and complications were not observed. Eighteen patients received either high-, mid-, or low-dose SVF for knee OA treatment [162]. Findings revealed that the high-dose injection of 1.0 × 10^8^ ADMSCs improved function and decreased pain without causing adverse effects. Infrapatellar fat pat derived ADMSCs were injected into knee joints of 18 patients [163]. Pain reduced and knee functions including MR findings improved in these patients. The same group previously published good outcomes for 25 knee OA patients to whom 1.9 × 10^6^ cells were injected [164]. A draw-back of this procedure could be the surgical removal of the infrapatellar fat pad. ADMSCs were also used in two patients to treat osteonecrosis of the femoral head [90,165]. More recently, 57 patients were treated with AD-cSVF and followed up for 12 months [166]. T2 mapping using MR revealed better values than preoperative scores. In another study [167], 84 patients were retrospectively reviewed. Swelling was a finding in 7% of the patients immediately after injection. Pain decreased and function improved in those patients with time. The effectiveness of the intra-articular injection of SVF could be cell-dose dependent [168]. The higher dose had better outcomes in 60 patients. Magnetic resonance findings of these patients however did not change. In another recent study [169], however, improvements in MR findings were reported in 53 knees of 47 patients who had grade 2 and 3 OA of the knee at the 12-month follow-up. At the 6 [170] to 12 month [171] follow-up, patients with good outcomes were confirmed by 2 [172] to 5 [173] year follow-up studies.

Medical devices for the preparation of AD-tSVF are summarized in Table 5 [85,174]. These devices are safely used in plastic, reconstructive and aesthetic surgery [175]. They are also frequently used to treat Achilles tendon injuries, rotator cuff ruptures of the shoulder joint, hand flexor tendon injuries, and osteochondral defect management. Their molecular action mechanism in articular cartilage is however very little known [176].

## 5. Non-Enzymatic tSVF Techniques and Technologies

### 5.1. Focus on Membrane Properties

The cell strainers made of different types of nylons and with the pore sizes of 40, 70, and 100 μm are commonly used in SVF isolation (https://www.sigmaaldrich.com/TR/en/substance/corningcellstrainer1234598765; accessed on 11 September 2022) [242]. Different SVF isolations involving the use of these filters are exemplified below. An optimized method for adipose SVF isolation was developed and applied in fat grafting [243]. For this purpose, the adipose tissue was grounded into an erosive shape and digested with collagenase. Following centrifugation, the pellets were suspended in DMEM and passed through a 100 μm strainer as a strong nylon mesh with 100 μm pores. Stem cells, tissue-derived cells, and cancer cells can be also filtered using this kind of filters, and they also have a function of filtering agglutinative proteins produced in inactivation serum. Isolated SVF was mixed with a fat graft and transplanted into mice in that study. The adipose tissue of a SVF co-transplanted group exhibited higher VEGF expression. SVF co-transplantation also inhibited adipose cell apoptosis. A novel method was also developed for the optimization of autologous adipose tissue recovery with extracellular matrix preservation [244]. After enzymatic digestion of fresh and lipocell-processed adipose tissue, SVF was isolated. The sample was centrifuged, and the pellet was resuspended, and the suspension was passed through 100 and 70 μm cell strainers, washed by saline solution. The combination of dialysis and brushing of the Lipocell procedure retained the cellular contents of the adipose tissue. The quantification of viable cells normalized for the adipose tissue weight showed no differences among the three filters with the pore sizes of 15, 20, and 50 μm. The yield of ADMSCs exhibited no significant change when comparing the filters.

A specific system and a new method for the automated isolation of SVF from adipose tissue lipoaspirate were proposed by SundarRaj et al. [245] SVF isolation was performed from lipoaspirate after enzymatic digestion. The digest was centrifuged, and the supernatant containing adipocytes was removed, and the pellet containing SVF was washed and filtered through a 100 μm cell strainer. In the referred study, a closed, automated system to process up to 500 mL lipoaspirate was developed using cell-size-dependent filtration technology. The yield of SVF obtained by automated tissue digestion and the filtration was almost the same to that obtained by manual isolation. The viability of the cells obtained by both methods was higher than 90%. Breast reconstruction was performed using enhanced SVF fat grafting [246]. Celution and Medikhan enzymatic systems, Fatstem and Mystem systems, and the mechanical separation system for SVF isolation were examined in terms of their clinical efficacy [242] in the treatment of soft-tissue defects in plastic and reconstructive surgery and knee OA. The SVF cell population from mature adipocytes and the extracellular matrix was separated by enzymatic digestion and subsequent centrifugation. The SVF cell population by centrifugation and subsequent filtration of the solution obtained through 0.2 μm filter was also obtained. In enhanced SVF-treated patients treated with cells obtained by the Celution system, 63% ± 6.2% maintenance of contour restoring was observed after 1 year (39% ± 4.4% of control group). In the patients treated with SVF obtained by the Medikhan, Fatstem, and Mystem systems, a lower maintenance of contour restoring values was obtained.

Clinical indications of mechanically isolated SVFs were also reviewed [133]. In the reviewed studies, different approaches were tried for SVF isolations. In the modified nano-fat approach, the emulsification step was performed using filter connectors or FFLL connectors. Following this stage, the filtration was made using superfine filters or filters with large pores between 400 and 600 μm [133]. In the last stage, the centrifugation was applied for the isolation of SVF. In the Tonnard’s nano-fat approach, the filtration with the Nano Transfer filter (500 μm) was used after the emulsification stage. In the SVF isolation with the FastKit system, the filtration (120 µm) and centrifugation stages were used [133]. The harvest and processing techniques for fat grafting and adipose stem cell isolation were compared [247]. The fat was harvested both by suction-assisted lipoaspiration and ultrasound-assisted lipoaspiration. The samples were then filtered using filters with two different pore sizes. Filtrands and filtrates were injected into athymic nude mice. Ultrasound-assisted lipoaspiration released slightly more oil than suction-assisted lipoaspiration. The fluid and oil were effectively removed by filtration with either a 500 or 800 μm pore size.

Adipose-derived SVF was processed with different systems for the treatment of knee osteoarthritis. A pilot study on cell proliferation and clinical results was performed [248]. Three processing systems (micro-fragmentation, filtration, or slow centrifugation) were applied to investigate cell proliferation in vitro and clinical results of intraarticular injections for the treatment of knee OA. The patients treated with SVF, obtained by the micro-fragmentation, exhibited better outcomes with a mean improvement in the symptomatology higher than that found in patients treated with the filtration or slow centrifugation system. The processing of lipoaspirate for autologous fat grafting by the roll, spin, wash, or filter was reviewed [249]. Randomized controlled trials, clinical trials, and comparative studies comparing at least two of the following techniques, namely, decanting, cotton gauze rolling, centrifugation, washing, filtration, and SVF, were taken into account. Nine articles were evaluated based on inclusion and exclusion criteria. Five of them compared established processing techniques (i.e., decanting, cotton gauze rolling, centrifugation, and washing), and four publications evaluated newer proprietary technologies, including washing, filtration, and/or methods to isolate SVF. The isolation of adipose-derived SVF cells was made using a novel point-of-care device [250]. The closed systems, namely, GID SVF-1 and GID SVF-2, are disposable, scalable cellular isolation devices designed for isolating the human adipose-derived stromal vascular fraction (AD-hSVF) from lipoaspirates. The results showed that adipose-derived AD-hSVF can be safely obtained using both devices and standardized methods, providing cells that were free of bacterial contaminants. The systems also allowed the selective capture of tissue fragments in an inner mesh filter compartment, while waste fluid was immediately aspirated into a waste container. The reference method for SVF isolation from lipoaspirate was compared with three medical devices: GID SVF-1, Puregraft, and Stem.pras [185]. The results demonstrated that all the devices allowed the production of SVF cells with a consistent yield and viability in shorter time than the reference method. Expanded cells from all protocols exhibited no significant differences in terms of phenotype, proliferation capabilities, differentiation abilities, and genetic stability.

### 5.2. Flow-Cytometry Analysis

**Figure 4:**
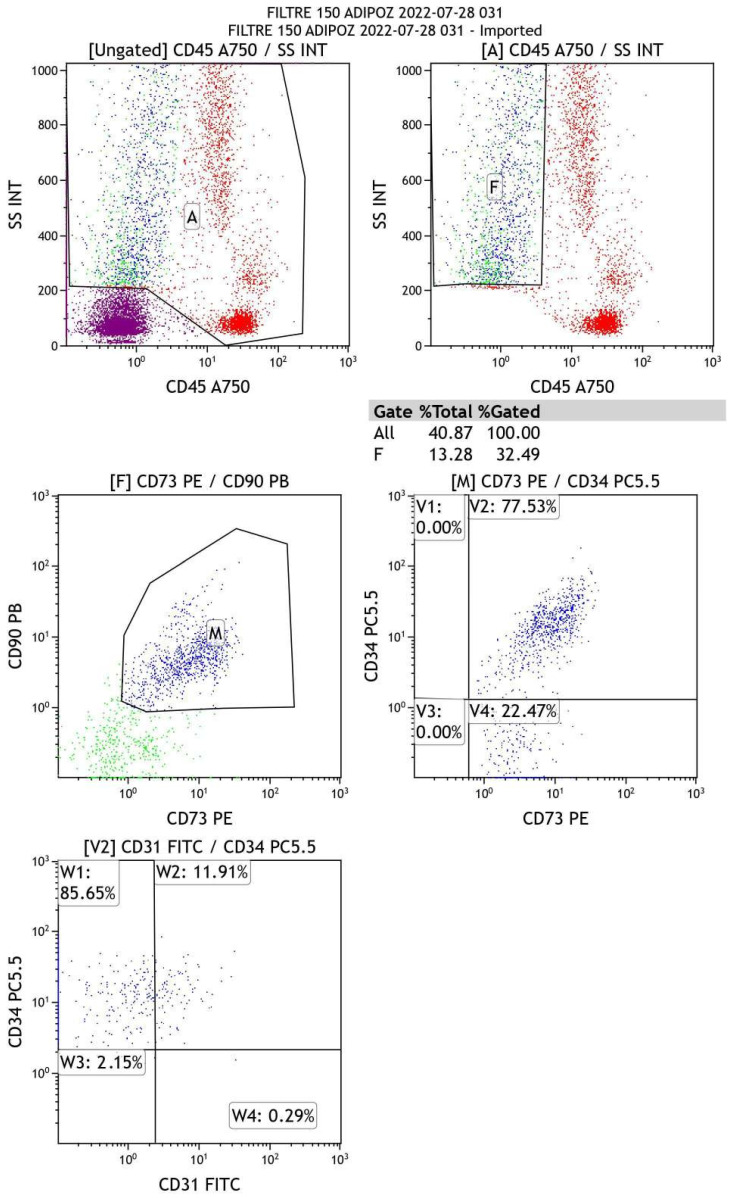
Flow cytometry analysis for AD-tSVF after filtration reveals cells in the composition are of stem cell nature.

We preliminarily evaluated the cellular outcome of an AD-tSVF medical device. A total of 60 mL autologous abdominal fat tissue was harvested from the abdominal subcutaneous tissue by liposuction using a Trans-luminescence solution. The lipoaspirate was washed with 60 mL phosphate-buffered saline (PBS) (Biological Industries, Beit-Haemek, Israel) to create a single-cell suspension that was centrifugated for 5 min at 1000× *g* to collect the cellular phase as a pellet. Once isolated, characterization of the cells was accomplished through a multi-color flow cytometry (Navios Flow Cytometer, Beckman Coulter, Indianapolis, IN, USA) device. The surface marker antigen expression panel was in agreement with IFATS and ISCT. The immunophenotypic analysis was to confirm the mesenchymal nature of isolated cells. The following fluorochrome-labeled monoclonal antibodies were used for AD-tSVF cell analysis. These were CD31-FITC, CD34-PC5,5, CD73-PE, CD90-PB, and CD45-A750 (Backman Coulter, USA). The CD31- or platelet endothelial cell adhesion molecule (PECAM-1) is normally found on endothelial cells, platelets, macrophages and Kupffer cells, granulocytes, lymphocytes, megakaryocytes, and osteoclasts. The CD34- cell-cell adhesion molecule, a marker for pluripotent stem cells, is expressed on all hematopoietic progenitor cells. The CD73- Ecto-5′-nucleotidase takes part in adipogenic and osteogenic differentiation. The CD90- that is also named as Thy-1 engages in adipogenic differentiation as well as adipose tissue homeostasis and metabolism. Associated with multipotent progenitor activity, the CD45- or protein tyrosine phosphatase receptor type C (PTPRC), leukocyte common antigen (LCA), and panhematopoietic marker are expressed on all human white blood cells. Markers were used in combination with ViaKrome (Beckman Coulter, Indianapolis, IN, USA), which determines cell viability, excluding debris and dead cells induced by the isolation protocol. Cells were incubated with the specific mAbs for 15 min. At least 105 cells were acquired from each sample. The software Navios EX, Navios (Beckman Coulter, Indianapolis, IN, USA) was used to create dot plots and to calculate the cell composition percentages according to the profile of the surface marker expressions. Data were analyzed in the Kaluza 2.1 software package (Beckman Coulter, Indianapolis, IN, USA). The graphs were prepared in GraphPad Prism (Figure 4). Findings revealed that AD-tSVF cells express MSCs’ nature.

## 6. Conclusions

Cells and fractions derived from the adipose tissue are used to regenerate musculoskeletal and soft tissues after minimal or to some extent maximum manipulation [192]. AD-tSVF as an example is also used in bone tissue engineering [2,251,252]. Adipose stem cells were encapsulated with a hydrogel for injectable cartilage regeneration [253]. They also stimulate the proliferation rates of human tendon cells [192]. Such regenerative treatments are currently used to enhance surgical outcomes [69] including the healing of high tibial osteotomy [254]. Cells and tissue fragments are expected to stimulate and/or maximize the intrinsic regenerative capacity including the joint cartilage [255]. We however know little how they function. The proteomic, lipidomic, and metabolomic pathways of stimulations is an active research area [256].

Recent systemic reviews and a meta-analysis [53,76,85,97,110,132,133,257,258,259,260,261,262] summarized the state of micro- and nano-fragmented fat tissue grafts, AD-cSVF, AD-tSVF, and ADMSCs. Here, we focused on autologous AD-tSVF applications in knee OA obtained from abdominal fat by liposuction. Meng et al. [257] listed the study design, interventional details, and outcomes of clinical studies that used autologous and allogenic ADMSCs obtained from the infrapatellar fat pad, gluteus, and abdomen. The lowest and the highest cell counts were 1.2 × 10^6^ and 1.0 × 10^8^ cells per mL, respectively. A total of 7 out of 18 studies had a control group, and 11 studies were case series. A total of 14 out of the 19 studies treated knee OA patients, and the lowest and highest number of patients ranged between 8 and 15, respectively. The mean age of patients in these studies ranged from 52.0 ± 8.4 to 64.6 ± 4.8 years. Follow-up lasted between 12 and 36 months, and serious adverse events were not observed in most of these studies. Pain, function scoring, and the second-look arthroscopic evaluation were the most common semi-quantitative outcome measures. Scoring by ultrasound, synovial fluid profiling, and MR were used in five of these reported studies. One study by Koh in 2016 [150,263] presented histological outcomes. The review of Agraval et al. [258] evaluated the study characteristics of ADMSCs and SVF clinical applications in knee defects. The repetition of ADMSCs’ application ranged between 1 and 3 in the included 11 studies. The number of patients ranged between 13 and 16, and the mean age ranged between 54.6 and 74.5 years in the SVF-administered 7 studies. The Kellgren-Lawrence Classification of included studies were between 0 to IV. Three out of the seven SVF administered studies used collagenase for preparation. Kunze et al. [85] listed enzymatic and mechanical ADMSCs’ products. They were also reviewed by Ude et al. [264]. Mechanical separation systems used centrifugation, syringe emulsification, electromagnetic vibration, and filtration. They also listed and commented on the knee OA patients’ results. The review of Ghiasloo et al. [133] focused on AD-tSVF, which was used in OA knees. The number of patients and knees ranged between 17–35 and 20–48, respectively. Biazzo et al. [97] listed four SVF and two ADMSCs studies. The mean age of patients ranged between 38.8 and 63.5 years. The follow-up period ranged between 6 and 27 months. Torres-Torillas et al. [76] covered seven studies that used subcutaneous abdominal fat for autologous ADMSCs’ isolation to treat chondral injuries. Mehranfar et al. [259] published SVF application studies. A total of 8 out of 11 studies combined PRP with SVF. Gentile et al. [260] comparatively analyzed five non-enzymatic procedures that used micro-fragmentation, filtration, washing, and purification. Cho et al. [110] listed scoring systems for assessing knee function after cartilage regeneration. They also summarized ADMSCs used for knee OA treatment. Van Dongen et al. [132] summarized the duration, cost, cell yield, and procedure characteristics of cSVF and tSVF. Roffi et al. [261] published details of nine SVF clinical papers. All papers combined PRP with SVF. Pak et al. [53] determined clinical studies on cartilage defect treatment with ADMSCs and SVF. Seven publications were on knee OA, and results were promising between 6 and 18 months. The same group [262] listed SVF treatment for orthopedic applications. Three of the listed studies were on knee pain management. Another recent review compared clinical studies of ADMSCs, bone marrow MSCs, and umbilical cord MSCs [265]. Another recent review [266] listed 12 clinical trials that used SVF in their patients. Minimally invasive interventions to the joint are suggested to overcome the limitations for OA treatment [267]. A recent review [268] highlighted that SVF was more effective than bone marrow autologous cells for knee pain. Magnetic resonance quantification after the treatment of knee OA with SVF is enhancing in recent studies [269].

We are currently improving our knowledge on the transition of treatment modalities from micro-and nano-fragmented fat tissue grafts to AD-cSVF, AD-tSVF, and ADMSCs. The number of patients in the follow-up studies is increasing [65]. There is a variation for the mean age of application in knee OA patients. Their stage of application also varies largely. The number of cells during applications is not standardized. A repetition of injections could change from a single to triple in practice. There are various products in the market, but the duration of centrifugation, filtration, washing, and purification in non-enzymatic procedures is not determined. Concomitant treatments could change the outcome among studies. It would be difficult whether the outcome is directly related to SVF application or concomitant procedures. Evaluations of outcomes were mostly by pain and function scoring. Few studies evaluated their patients by second-look arthroscopy. Advanced techniques including ultrasound and MR were included in newer studies. The optimum pressure applied to the tissue and cells during filtration and purification for AD-tSVF should be determined.

AD-tSVF could avoid possible complications of AD-cSVF or ADMSCs’ separation techniques. The duration of centrifugation, filtration, washing, and purification in AD-tSVF procedures is however not determined. Repetition and pressure applied on the fat tissue during mechanical separation and filtration need additional experimentation. We recommend minimal manipulations of the harvested fast tissue and use gravitational force only for filtration. The size of filters and their properties should also be well defined. Most of the clinical applications were after the preparation of the joint cartilage by arthroscopic irrigation, micro- or nano-fracturing of the subchondral bone. Knee OA on the other hand contains a pathology of the whole joint and joint-related structures. The paracrine effect of AD-tSVF is therefore quite important. AD-tSVF is frequently applied together with PRP to decrease inflammation and increase the paracrine effect. The number of injection repetitions and durations is however not determined. The number of cells during each application is studied; however, molecular and metabolic outcomes after applications were mostly on safety, pain, and function. Advanced quantitative outcome measures such as ultrasound, MR, and synovial fluid assessment was recently introduced into studies. Future studies that will clarify these issues will help to improve the quality of life of our knee OA patients.

## Figures and Tables

**Table 1:** Diseases in which Stromal Vascular Fraction Obtained from Adipose Tissue can be Applied.

Coronary heart disease
Peripheral vascular diseases (Burger’s Disease)
Dermatopathy
Chronic wounds (Pressure ulcers)
Ischemia
Fistula
Liver fibrosis
Lipodystrophy
Radiation-induced ulcers
Alopecia
Osteoarthritis (OA)
Rheumatoid Arthritis (RA)
Systemic lupus erythematosus (SLE)
Diabetic foot ulcers
Breast cancer-related lymphedema
Crohn’s disease
Erectile dysfunction
Type 2 diabetes mellitus
Multiple sclerosis (neurodegenerative diseases)
Chronic obstructive pulmonary disease (COPD)
Nonunion fractures (High tibial osteotomy etc.)
Micromastia
Fat and skin grafts
Systemic sclerosis
Rotator cuff injury
Tendinopathy (Achilles tendinitis)
Osteochondral defects

Note: The listed diseases are registered with clinicaltrials.gov (accessed on 12 August 2022) and are in the clinical investigation phase.

**Table 2:** Effect of Stromal Vascular Fraction on Tissues.

Anti-inflammatory	Reduces tissue swelling (edema).
Anti-apoptotic	Reduces and stops programmed cell death.
Anti-fibrotic	Prevents tissue adhesions.
Increasing of TIMPs-1, -3, and -4 metalloproteinases	Provides tissue balance (Homeostasis).
Inhibition of MMP-1, MMP-3, MMP-13, and MMP-28 metalloproteinases	Provides tissue balance (Homeostasis).
Increasing of ADAMTS-4 and -5	Provides tissue balance (Homeostasis).
Regulation of pro-inflammatory molecules	Decreases IL-1b and IL-6 levels.
Triggering of IL-1Ra	Reduces the catabolic effect of IL-1.
Hyaline cartilage ECM	Increases GAG level.

**Table 3:** Advantages and Disadvantages of Enzymatic and Mechanical Stromal Vascular Fraction Separation Methods.

	Enzyme Decomposition	Mechanical Separation
Initial amount of adipose tissue	300 mL ↑	60 mL ↓
Incubation	(+)	(−)
Washing	(+)	(−)
Centrifuge	(+)	(−)
Device	(+)	(−)
Disposable consumable	(+)	(+)
Reliability	(+/−)	(+)
Bacterial contamination	(+/−)	(−)
Enzyme-related side effects in tissue	(+)	(−)
Implementation cost	↑	↓
Duration of implementation	2 h ↑	1 h ↓
Number of cells	↑	↓
Cell surface marker	↓	↑

**Table 4:** Steps of Stromal Vascular Fraction Separation [98].

	Conventional	Modified Approach
Obtaining adipose tissue	- Abdominal fat - Reusable Sorenson type lipoaspiration cannula - Klein’s Translumination solution: Modified Klein solution (500 mL isotonic, 20 mL lidocaine, 2% epinephrine, 2 mL bicarbonate) - 50 mL Luer-Lock syringe	- Abdominal fat - Disposable/Re-usable Coleman style cannula - Klein’s Translumination solution: Modified Klein solution (500 mL isotonic, 20 mL lidocaine, 2% epinephrine, 2 mL bicarbonate) - 50 mL Luer-Lock syringe
Mechanical separation/shredding	- Shredding of tissue by shaking with glass ball (shaking time and strength depend on the user)	- Separation by the effect of gravity in a screw form mechanical separator at standard power and time.
Pre-filtration	- Polyethylene filtration in a 100 micrometer porous polyethylene bag	-Filtration with the effect of gravity in the 100 micrometer porous device whose base will be supported by a metallic or polymeric cage.
Washing	(−)	- Washing in the device
Final filtration	-Filtration on 10 micrometer porous polyethylene filters in 10 mL syringes (50 repetitions?)	- Final filtration with the rise of adipose tissue and SVF to the solution surface in serum within the device.
Collection of SVF/adipose tissue	- Available in an equivalent system	-Proximal adipose tissue and SVF separation reservoir.
Cell counting and characterization	- Cell counting, determination of viability, determination of cell characteristics, and histochemical identification	- Cell counting, determination of viability, determination of cell characteristics, and histochemical identification

**Table 5:** Commercial Medical Products for AD-tSVF Preparation (accession date to the web sites was 22 August 2022).

Product	Company	Article
Cha-Station	Somnotec http://www.somnotec.net	[119]
Octagone D200	Endecotts Ltd. https://www.endecotts.com	[177]
AdiPrep	Harvest http://www.harvest.co.kr/clinician/clinician-home/adiprep/advantages/quality.html	[178]
Lipokit	Medi-Khan http://www.medikanint.com	[119,179,180]
Puregraft 250	Puregraft LLC http://www.puregraft.com	[181,182,183,184,185,186,187,188,189,190,191]
Lipogems	Lipogems http://understandlipogems.com	[72,80,111,140,174,192,193,194,195,196,197,198,199,200,201,202,203,204,205,206,207,208,209,210,211,212,213,214,215,216,217,218,219,220,221]
MyStem	MyStem LLC https://mystem.eu/	[222,223,224,225]
LipoStem	Biopsybell https://www.biopsybell.com/products/ortho-biologics/lipo-stem-duo-adipose-tissue-admsc-microfragmentation-kit/	
Arthrex SVF	https://www.arthrex.com/orthobiologics	[226,227,228]
Adinizer	BSL http://biosl.com/?ckattempt=1	[229,230]
Microlyser	TLab https://tlab.com.tr/en/products/microlyzer-svf-kit/	[231]
SEFFIE	Advanced-Maes http://www.advanced-maes.com/	[232]
LIPOCUBE	STEMC https://lipocube.com/	[233,234]
Fastkit (Fastem)	CORIOS Soc. Coop. https://www.corios.it/	[235]
Q-Graft	Human Med AG https://www.humanmed.com/en/products/q-graft/	[236]
Tulip Nanotransfer	Tulip Medical https://tulipmedical.com/	[233,237]
Lipocell	Tissyou https://www.tissyou.com/portfolio_page/lipocell/	[238]
LipiVage	Genesis Biosytems https://www.genesisbiosystems.com/lipivage-system-autologous-fat-transfer/	[239,240,241]

## Data Availability

The data presented in this study is openly available.

## Abbreviations

AD	Adipose Derived
MSCs	Mesenchymal Stem Cells
SVF	Stromal Vascular Fraction
AD-tSVF	Adipose Derived tissue Stromal Vascular Fraction
AD-cSVF	Adipose Derived cellular Stromal Vascular Fraction
AD-hSVF	Adipose Derived human Stromal Vascular Fraction
PRP	Platelet Rich Plasma
ECM	Extracellular Matrix
HA	Hyaluronan
GAG	Glycosaminoglycan
CS	Chondroitin Sulfate
KS	Keratan Sulfate
OA	Osteoarthritis
BMI	Body Mass Index
YLD	Global Years Lived with Disability
DALYs	Disability Adjusted Life Years
USD	United States Dollar
NK Cells	Natural Killer Cells
IFN	Interferon
TNFα	Tumor Necrosis Factor alpha
TGFb	Transforming Growth Factor beta
IGF-1	Insulin-like Growth Factor 1
HIF	Hypoxia-Inducible Factor
MMPs	Matrix metalloproteinases
ADMSCs	Adipose Derived Mesenchymal Stem Cells
IL	Interleukin
MSCs	Mesenchymal Stem Cells
WHO	World Health Organization
OP	Osteoporosis
FDA	Food and Drug Administration
IFATS	The International Federation for Adipose Therapeutics and Science
ISCT	The International Society for Cell & Gene Therapy
ATMP	Advanced Therapy Medicinal Products
ITR2	Injectable Tissue Replacement and Regeneration
KFDA	Korean Food and Drug Administration
PDGF	Platelet-Derived Growth Factor
VEGF	Vascular Endothelial Growth Factor
FGF	Fibroblast Growth Factor
FGFR	Fibroblast Growth Factor Receptor
TIMPs	Tissue Inhibitors of Metalloproteinases
ADAMTS	A Disintegrin and Metalloproteinase with Thrombospondin Motifs
MR	Magnetic Resonance
CaCl2	Calcium Chloride 2
VAS	Visual Analogue Scale
WOMAC	Western Ontario and McMaster Universities Arthritis Index
KOSS	The Knee Osteoarthritis Scoring System
PTPRC	Protein Tyrosine Phosphatase Receptor type C
LCA	Leukocyte Common Antigen
EPISER	Prevalence of Rheumatic Diseases in Adult Population in Spain Study
Arg1	Arginase 1
PBS	Phosphate-Buffered Saline
HLA	Human Leucocyte Antigen
DMEM	Dulbecco’s Modified Eagle Medium
PECAM-1	Platelet Endothelial Cell Adhesion Molecule
FFLL	Female-to-Female Luer-Lock
MMPs	Matrix Metalloproteinase
COMP	Cartilage oligometric matrix protein
GDF-11	Growth differentiation factor-11
